# Control of Klebsiella pneumoniae Infection in Mice by Using Dissolving Microarray Patches Containing Gentamicin

**DOI:** 10.1128/AAC.02612-18

**Published:** 2019-04-25

**Authors:** Aoife M. Rodgers, Maelíosa T. C. McCrudden, Aaron J. Courtenay, Mary-Carmel Kearney, Katherine L. Edwards, Rebecca J. Ingram, Jose Bengoechea, Ryan F. Donnelly

**Affiliations:** a School of Pharmacy, Medical Biology Centre, Queens University Belfast, Belfast, United Kingdom; b Wellcome-Wolfson Institute for Experimental Medicine, School of Medicine, Dentistry & Biomedical Science, Queens University Belfast, Belfast, United Kingdom

**Keywords:** *Klebsiella pneumoniae*, gentamicin, microarray, transdermal delivery

## Abstract

Using a murine model of Klebsiella pneumoniae bacterial infection, we demonstrate that gentamicin dissolving microarray patches, applied to murine ears, could control K. pneumoniae infection. Mice treated with microarray patches had reduced bacterial burden in the nasal-associated lymphoid tissue and lungs compared with their untreated counterparts. This proof of concept study represents the first published data on the *in vivo* delivery of the antibiotic gentamicin via dissolving microarray patches, resulting in the control of bacterial infection.

## Abstract (precis)

Using a murine model of Klebsiella pneumoniae bacterial infection, we demonstrate that gentamicin dissolving microarray patches, applied to murine ears, could control K. pneumoniae infection. Mice treated with microarray patches had reduced bacterial burden in the nasal-associated lymphoid tissue and lungs compared with their untreated counterparts.

## INTRODUCTION

Neonatal infections, including pneumonia and sepsis, remain a significant cause of mortality and morbidity, with an estimated 3 million neonatal deaths occurring every year worldwide (1). Neonates born in low- and middle-income countries are at greatest risk of mortality due to bacterial infections because of limited access to hospitals, facility-based care, or lifesaving antibiotics (2). In response to this, the World Health Organization (WHO) has provided guidelines for managing possible serious bacterial infections (PSBI) in young infants when referral to a hospital is not possible. Treatment includes intramuscular (i.m.) gentamicin (GEN) in combination with oral amoxicillin (AMX) (3).

GEN is a potent aminoglycoside antibiotic with bactericidal activity against Gram-negative bacteria and is widely utilized due to its efficacy and low cost (4). Similar to other aminoglycosides, GEN has a narrow therapeutic index and has the potential for ototoxicity and nephrotoxicity (5, 6). GEN is excreted in the kidneys primarily by glomerular filtration and has a short plasma elimination half-life in healthy individuals presenting with normal renal function (7). In neonates and young children, GEN half-life can vary according to weight, and thus, careful dose calculation based on infant weight is necessitated (8, 9). As a consequence of this, well-resourced settings have implemented therapeutic drug monitoring of GEN serum levels to reduce the incidence of toxicity (4). However, in outpatient resource-poor settings, this regimen has challenges and many neonates do not receive appropriate treatment (10). For those that do receive antibiotics, drug levels are unmonitored and first-line care is often provided by those lacking specialist pediatric training, often resulting in dose miscalculations and subsequent toxicity. As GEN must be delivered by i.m. injection, health care providers require access to safe injection supplies and sharps disposal, which is often unavailable in low-resource settings (11).

Based on the aforementioned challenges, it is evident that novel, simplified approaches are warranted to expand access to lifesaving antibiotics in this population group. Accordingly, we have developed dissolving microarray patches (MNs) (also known as microneedles) for transdermal delivery of GEN (7). MNs are minimally invasive devices that consist of an array of microscopic needles attached to a base support (12). Upon insertion into the skin, the needles create microscopic holes, bypassing the stratum corneum barrier and subsequently delivering drug contained in the MNs into the viable skin (reviewed in reference 13). MNs are typically fabricated such that they are short enough to avoid stimulation of dermal nerves, and therefore, they provide a simplified, painless method of drug delivery that is well accepted by human subjects (14, 15). MNs offer the possibility for GEN delivery by less-experienced personnel and easier logistics for supplying to remote areas (16). As MNs dissolve upon insertion in the skin, they eliminate the requirement for sharps disposal and avoid transmission of blood-borne infections through needlestick injuries (17). Our previous work has demonstrated the successful transdermal delivery of therapeutically relevant concentrations of GEN using dissolving MNs in an *in vivo* model (7). However, the *in vivo* efficacy of antibiotics delivered transdermally via MN to control bacterial infection has yet to be demonstrated.

**FIG 1:**
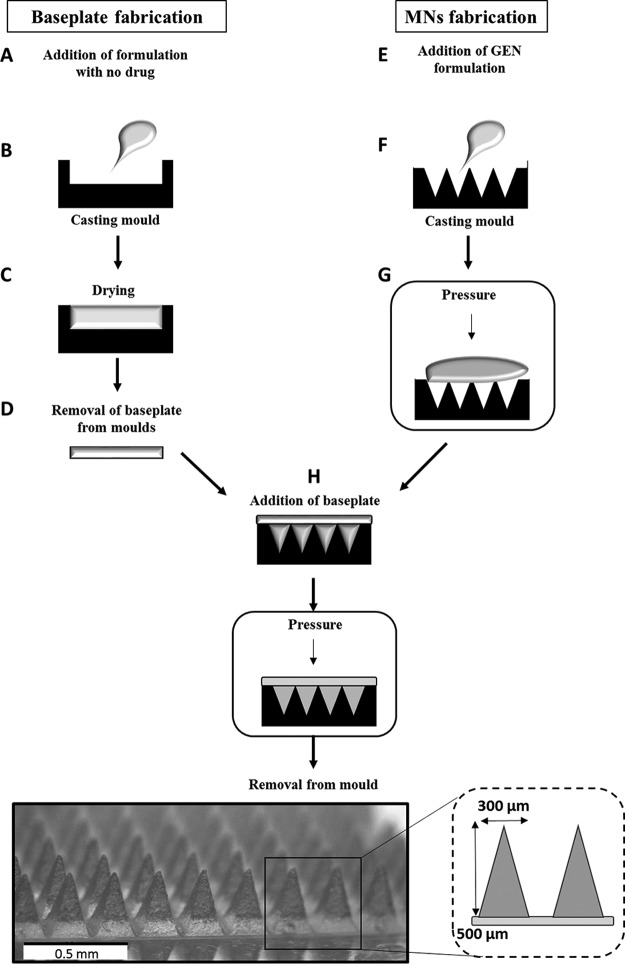
A schematic illustration outlining the steps involved in the manufacture of MNs containing GEN. MNs were prepared in two steps, namely, the fabrication of the baseplates which contained no drug and the fabrication of the needles which contained GEN. (A) Baseplates were prepared from 15% PVP (360 kDa) and contained no GEN. (B) The formulation (250 mg) was cast into MN molds devoid of needles, and (C and D) baseplates were dried for 24 h before being removed. For the MN formulation, (E) selected polymers and GEN (3.4% sodium hyaluronate, 250 to 400 kDa), in combination with 1% PVP (58 kDa) and 10% GEN sulfate, were mixed and sonicated for 4 h. (F) Following this, 25 mg of MN formulation was added to MN molds, and (G) a pressure (3 to 4 bar) was applied for 15 min to fill the molds. (H) The baseplates were then applied to the backs of the MN, and pressure was applied as previously described. The combined MNs were dried for 48 h before being carefully removed from the molds and microscopically analyzed to ensure complete formation.

In this study, we aimed to test the therapeutic efficacy of antibiotics delivered via MNs. MNs containing GEN were prepared utilizing simplified manufacturing processes, as previously described (Fig. 1) (12). MNs were formulated from aqueous blends containing 3.4% sodium hyaluronate, with molecular weight (MW) 250 to 400 kDa, in combination with 1% polyvinylpyrrolidone (PVP; 58 kDa; Sigma-Aldrich, Dorset, UK) and containing 10% GEN sulfate (Tokyo Chemical Industry UK Ltd., Oxford, UK). Upon insertion of MNs into skin, the drug content in the needles is delivered concurrently with MN dissolution. Some drug may also diffuse into the skin layers from the baseplate, thus, allowing for sustained drug release (18). GEN exhibits a concentration-dependent bactericidal effect with peak and trough serum concentrations that are therapeutically effective and nontoxic. Sustained delivery of GEN, with peak serum levels above 10 to 12 μg/ml and trough serum levels above 2 μg/ml, may be toxic (7, 19). Accordingly, we fabricated MNs in two steps to localize the GEN content to needles and, thus, prevent toxicity associated with sustained GEN delivery. Thus, the baseplates contained no GEN and were formulated from 15% PVP (360 kDa). The resultant MNs had heights of approximately 500 μm and base widths of approximately 300 μm, as confirmed by microscopic analysis (Fig. 1).

**FIG 2:**
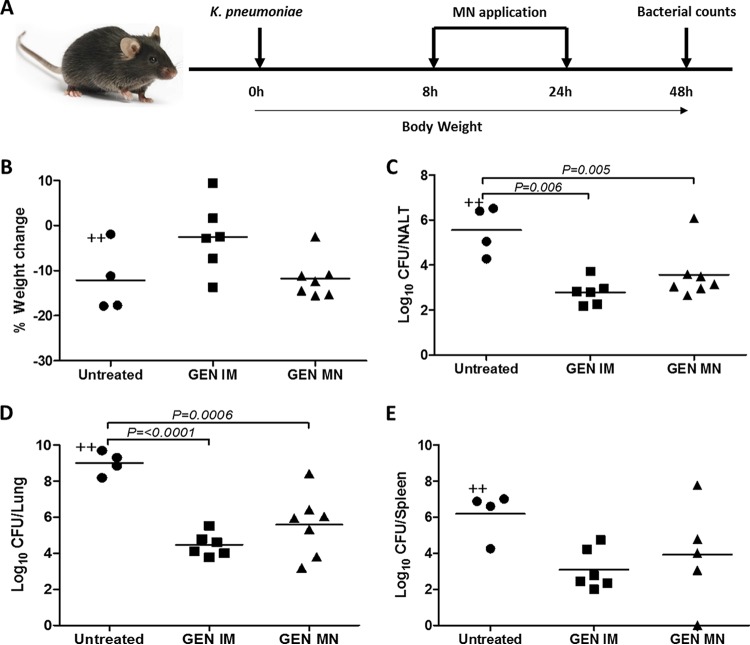
Dissolving MNs containing GEN control K. pneumoniae infection in mice. (A) A schematic representation of the treatment regime. Mice (C57BL/6), 8 to 10 weeks old, were infected intranasally with 10^5^ CFU K. pneumoniae bacteria. The mice were then either untreated (Untreated) or were treated with GEN delivered via i.m. (7.5 mg/kg of body weight in sterile water for injection and delivered via the thigh muscle of the hind limb) (GEN i.m.), or with dissolving GEN MNs (GEN MN) applied to the dorsal surface of each ear using micropore tape for 24 h, after which MNs were removed and replaced with a new GEN MN. (B) Body weights of mice were monitored throughout the course of the infection. At 48 h postinfection, mice were sacrificed and bacterial counts were determined in the (C) nasal-associated lymphoid tissue (NALT), (D) lungs, and (E) spleens. CFU counts for individual mice are shown with solid lines corresponding to mean values, *n* =6-7 mice/group, and “+” indicates an animal that died. Statistical significance was determined using Prism 7 (GraphPad) software using a one-way ANOVA, followed by correction for false discovery rate via the two-stage step-up method of Benjamini, Krieger, and Yekuutieli.

We subsequently probed the capacity of GEN MNs to induce therapeutic effects *in vivo*. We tested the GEN MNs in a Klebsiella pneumoniae murine model of pneumonia. K. pneumoniae is one of the most important Gram-negative pathogens associated with a wide spectrum of infections, including pneumonia, intra-abdominal infections and bloodstream infections (20, 21). GEN is a clinically relevant antibiotic treatment against K. pneumoniae, and therefore, this bacteria was selected as a model pathogen (22–24). Mice were infected with a live culture of K. pneumoniae (ATCC 43816) delivered intranasally (10^5^ CFU per mouse in 30 μl of endotoxin-free phosphate-buffered saline [PBS]), and this results in dissemination 24 h postinfection. The inoculum was plated for confirmation of bacterial number/load. Following this, mice (*n* = 6 to 7/group) were treated as per the schematic in Fig. 2A. In short, 8 h post-K. pneumoniae infection, GEN MNs were applied to the dorsal surface of each murine ear and held in place using micropore tape for 24 h, after which they were removed and replaced with additional MNs. GEN delivered i.m. to the thigh muscle of the hind limb was included as control. At 48 h postinfection, mice were sacrificed and the organs were harvested for analysis of bacterial burden. Body weights were monitored over the course of infection; however, no significant differences between groups were observed 48 h postinfection (Fig. 2B). As demonstrated in Fig. 2C, mice which received GEN i.m. or GEN MNs exhibited a greater capability to control infection, as evidenced by reduced numbers of CFU in the nasal-associated lymphoid tissue (NALT) and lungs. A significant reduction in CFU was observed in the NALT (*P* = 0.0053) and lungs (*P* = 0.0006) of mice treated with MNs compared with their untreated counterparts, demonstrating the *in vivo* activity of GEN delivered via MN. Specifically, application of GEN MN resulted almost a two-log reduction in the number of CFUs in the NALT and a 3.4-log reduction in lung CFU. A one-way analysis of variance (ANOVA), followed by correction for false discovery rate, was used for determination of statistical significance. A greater spread of results was evident in mice receiving GEN MNs than that of mice receiving GEN i.m., and this is likely attributable to the variability in the MNs manufactured under small-scale laboratory conditions. All experiments were performed in accordance with the UK Home Office and approved by the Queens University Belfast Ethical Review Committee.

In conclusion, the results presented herein collectively demonstrate that MNs containing GEN effectively control K. pneumoniae infection in mice. While further studies are warranted to demonstrate complete clearance, this is the first reported study utilizing MNs for the treatment of bacterial infection. MNs may be a potentially viable delivery platform for antibiotic delivery, offering the possibility to expand access to lifesaving antibiotic treatment in low-resource settings. As MNs dissolve upon insertion in the skin, they circumvent the generation of sharps waste and associated transmission of blood-borne pathogens. Our ongoing efforts entail the optimization of GEN MNs to increase bioavailability and develop a thorough understanding of the pharmacokinetics and pharmacodynamics of GEN delivered via this route compared with that delivered i.m. In the era of increasing antimicrobial resistance, novel approaches for empirical therapy are necessitated and MNs may offer an ideal solution. Importantly, as MNs bypass the gastrointestinal microbiota, they also offer an alternative delivery option for antibiotics that are currently delivered orally and could potentially prevent dysbiosis of the gut microbiota.
